# Loop-tipped guidewire-assisted cannulation of disposable imaging catheter into appendiceal lumen for difficult endoscopic retrograde appendicitis therapy

**DOI:** 10.1055/a-2641-2086

**Published:** 2025-07-17

**Authors:** Yuhong Ren, Ping Wang, Wenguang Yang, Sichao Wen, Haiyong Long, Mingwen Guo

**Affiliations:** 1 Department of Gastroenterology, Qionglai Medical Center Hospital, Qionglai, China

Endoscopic retrograde appendicitis therapy (ERAT) is an emerging minimally invasive approach for the diagnosis and treatment of acute appendicitis 1 . The disposable imaging catheter provides direct visualization of the appendiceal lumen, enabling irrigation, fecalith removal, and stent placement 2 . Similarly to endoscopic retrograde cholangiopancreatography (ERCP), cannulation in ERAT remains challenging due to edematous or stenotic appendiceal orifices 3 . This report describes a novel technique using a loop-tipped guidewire to guide a disposable imaging catheter to the appendiceal orifice during difficult intubation, facilitating successful cannulation.

A 24-year-old man with a 5-day history of right lower quadrant pain declined surgery in favor of conservative management. Computed tomography showed appendiceal dilation (14 mm) with peri-appendiceal inflammation ( Fig. 1 **a** ). ERAT was proposed as an alternative therapeutic approach.

**Fig. 1:**
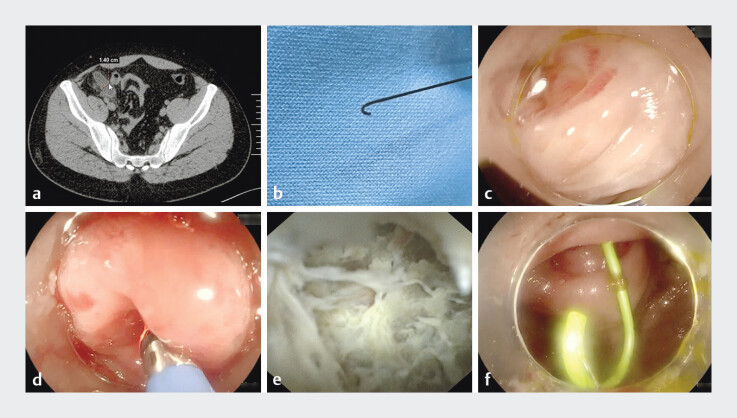
Loop-tipped guidewire-assisted cannulation of disposable imaging catheter into the appendiceal lumen. **a** Computed tomography showing dilated appendix (14 mm). **b** Loop-tipped guidewire modification. **c** Edematous appendiceal orifice on colonoscopy. **d** Successful catheter cannulation. **e** Endoscopic view of purulent debris. **f** Deployed single-pigtail stent.

A standard ERCP guidewire (Jagwire; Boston Scientific, Marlborough, Massachusetts, USA) was manually shaped to form a loop at its tip ( Fig. 1 **b** ). Colonoscopy identified a severely edematous and hyperemic appendiceal orifice ( Fig. 1 **c** ). The disposable imaging catheter failed to access the appendiceal lumen. The loop-tipped guidewire was advanced through the stenotic orifice using its rounded contour and hydrophilic coating to navigate mucosal folds ( Video 1 ). The disposable imaging catheter (ClearPath; Micro-Tech, Nanjing, China) was then threaded over the guidewire into the appendiceal lumen ( Fig. 1 **d** , Video 1 ). Direct visualization confirmed extensive purulent debris ( Fig. 1 **e** ), which was irrigated with metronidazole until clear effluent was observed. A 5 Fr × 5 cm single-pigtail plastic stent was then deployed for drainage ( Fig. 1 **f** ).

Loop-tipped guidewire-assisted cannulation of disposable imaging catheter into appendiceal lumen for difficult endoscopic retrograde appendicitis therapy.Video 1

The patient had immediate postoperative pain relief, with discharge on Day 1. The stent passed spontaneously by Day 4, and 2-week follow-up confirmed complete symptom resolution.

The loop-tipped guidewire displaces mucosa to create a catheter advancement track, minimizing trauma. Its hydrophilic coating and loop design reduce mucosal adhesion in tortuous/inflamed anatomy, guiding the imaging catheter to complete appendiceal cannulation. The simplicity, safety, and efficiency of the technique warrant broader clinical adoption.

Endoscopy_UCTN_Code_TTT_1AT_2AF

## Notes

Endoscopy E-Videos https://eref.thieme.de/e-videos
*E-Videos* is an open access online section of the journal *Endoscopy* , reporting on interesting cases and new techniques in gastroenterological endoscopy. All papers include a high-quality video and are published with a Creative Commons CC-BY license. Endoscopy E-Videos qualify for HINARI discounts and waivers and eligibility is automatically checked during the submission process. We grant 100% waivers to articles whose corresponding authors are based in Group A countries and 50% waivers to those who are based in Group B countries as classified by Research4Life (see: https://www.research4life.org/access/eligibility/ ). This section has its own submission website at https://mc.manuscriptcentral.com/e-videos .
